# Integrated Evaluation of Corneal Damage, Goblet Cell Remodeling and Inflammatory Response in a Murine Model of Environmental Dry Eye Disease (DED)

**DOI:** 10.3390/biomedicines14030693

**Published:** 2026-03-17

**Authors:** Alessandro Vitola, Gloria Astolfi, Chiara Tugnoli, Francesca Gobbo, Luca Lorenzini, Giuseppe Sarli, Piera Versura

**Affiliations:** 1 Department of Veterinary Medical Sciences (DIMEVET), Alma Mater Studiorum University of Bologna, Ozzano dell’Emilia, 40064 Bologna, Italy; alessandro.vitola2@unibo.it (A.V.); francesca.gobbo3@unibo.it (F.G.); luca.lorenzini8@unibo.it (L.L.); giuseppe.sarli@unibo.it (G.S.); 2 Interdepartmental Centre for Industrial Research in Health Sciences and Technologies—CIRI Health Sciences and Technologies, Alma Mater Studiorum University of Bologna, Ozzano dell’Emilia, 40064 Bologna, Italy; 3 Ophthalmology Unit, Department of Medical and Surgical Sciences (DIMEC), Alma Mater Studiorum University of Bologna, 40138 Bologna, Italy; gloria.astolfi2@unibo.it (G.A.); piera.versura@unibo.it (P.V.); 4 IRCCS AOU di Bologna, 40138 Bologna, Italy

**Keywords:** dry eye disease, desiccating stress, animal model, goblet cell remodeling, ocular surface inflammation, mucin alteration, controlled-environment chamber

## Abstract

**Background**: Dry Eye Disease (DED) is a multifactorial disorder characterized by tear film instability and ocular surface inflammation. Murine models based on environmental stress are widely used to mimic evaporative DED, although many focus on limited disease features. This study aimed to provide an integrated characterization of ocular surface alterations induced by chronic desiccating stress. **Methods**: Adult mice were housed in a Controlled-Environmental Chamber (CEC) with low humidity and increased airflow for up to 21 days and sacrificed after 14 or 21 days. Corneal damage was assessed by fluorescein staining. Conjunctival histology was evaluated for epithelial morphology, goblet cell (GC) size, and mucin composition. Complement fractions C3 and C5a were assessed by immunohistochemistry. Expression of inflammatory markers (Major Histocompatibility Complex, Class II, DR, HLA-DR; interleukin-1β, IL-1β; tumor necrosis factor-α, TNF-α) was quantified by Real-Time PCR (RT-PCR) in corneal and conjunctival epithelium. **Results**: Fluorescein staining revealed progressive corneal epithelial damage over time. Histological analysis demonstrated conjunctival epithelial alterations characterized by a significant reduction in GC size and in neutral mucin-positive GCs, consistent with mucin remodeling of the ocular surface epithelium. Increased epithelial deposition of complement fractions C3 and C5a was observed, while molecular analysis confirmed upregulation of inflammatory markers, including HLA-DR, IL-1β, and TNF-α. Collectively, these findings indicate that the model captures key pathophysiological components of DED. **Conclusions**: The CEC model reproduces major features of evaporative DED, including epithelial damage, GC remodeling, immune activation, and inflammation. As a non-invasive desiccating stress model, it represents a relevant experimental platform for studying ocular surface inflammation and for preclinical evaluation of therapeutic strategies.

## 1. Introduction

Dry eye disease (DED) is a multifactorial disorder of the ocular surface characterized by tear film instability, hyperosmolarity, inflammation, and neurosensory abnormalities, leading to ocular discomfort and visual disturbance [1,2]. According to the Tear Film & Ocular Surface Society Dry Eye Workshop (TFOS DEWS) II report, DED arises from dysfunction of the lacrimal functional unit and is sustained by a complex interplay between epithelial damage, immune activation, and environmental stressors [1]. Increasing evidence supports the concept of DED as a chronic inflammatory disease, in which ocular surface injury and immune-mediated mechanisms perpetuate a self-amplifying cycle of inflammation and tissue damage [2,3,4].

Animal models have played a central role in improving our understanding of DED pathophysiology and in supporting the development and preclinical assessment of therapeutic strategies. In addition to surgical excision and pharmacological inhibition approaches, alternative experimental strategies have been described, including obstruction of lacrimal gland ducts, which induces aqueous-deficient dry eye through mechanical impairment of tear secretion, and sleep deprivation-induced dry eye, which reproduces tear film instability and ocular surface inflammation associated with circadian disruption. These models further emphasize the multifactorial nature of DED and the diversity of mechanisms leading to ocular surface damage [5,6,7,8,9]. Among these, surgical removal of the lacrimal gland has been widely used to induce aqueous-deficient dry eye in rodents and has demonstrated reproducible reductions in tear production associated with corneal damage and ocular surface inflammation [10,11]. However, surgical models are invasive, irreversible, and predominantly reflect tear deficiency, thus only partially reproducing the multifactorial nature of human DED [5,12]. Moreover, the disruption of the lacrimal apparatus may limit the translational relevance of these models when evaluating therapies aimed at restoring tear secretion or modulating physiological tear dynamics.

In contrast, environmentally induced dry eye models have gained increasing attention due to their ability to mimic clinically relevant desiccating conditions, such as low humidity, increased airflow, and prolonged ocular surface exposure [13,14,15]. Environmental stress is a recognized risk factor for DED in humans, particularly in modern indoor settings characterized by air conditioning, screen use, and reduced blink rate [16]. Murine models of environmental dry eye allow induction of ocular surface disease without surgical manipulation or systemic pharmacological interference, preserving lacrimal gland integrity while eliciting hallmark features of DED, including tear film instability, epithelial damage, and inflammatory activation [13,14,17]. The mouse represents a particularly suitable species for such studies, owing to the availability of immunological tools, molecular assays, and well-characterized inflammatory pathways relevant to ocular surface disease.

Beyond tear film alterations, DED profoundly affects ocular surface tissues, including the corneal epithelium and the conjunctival GCs population. GCs are essential for mucin secretion and tear film stability, and their loss or phenotypic remodeling contributes to mucin deficiency and exacerbation of epithelial stress [18,19]. Experimental and clinical studies have demonstrated that desiccating stress induces GC dysfunction, including altered mucin expression and, in some models or disease stages, GC loss, with increased susceptibility of the ocular surface to inflammatory injury [20,21]. Concurrently, pro-inflammatory mediators such as interleukin-1 (IL-1), tumor necrosis factor-α (TNF-α), and matrix metalloproteinase-9 (MMP-9) are upregulated, promoting epithelial barrier disruption and immune cell recruitment [3,22,23,24]. Despite the recognized interdependence of epithelial damage, GC dysfunction, and inflammation, many experimental studies focus on isolated endpoints, limiting the ability to capture the integrated nature of ocular surface pathology in DED.

Therefore, the aim of the present study was to establish and characterize a non-surgical murine model of environmental dry eye, based on controlled desiccating stress, and to provide an integrated evaluation of corneal damage, GC remodeling, and inflammatory response. By integrating clinical, histological, and immunological outcomes, this study aims to determine whether the proposed model can reproduce early functional alterations of the ocular surface and reflect key features of the multifactorial pathophysiology of human DED, thereby supporting translational and preclinical research.

## 2. Materials and Methods

### 2.1. Materials

The following principal reagents and materials were used in this study: Isoflo (isoflurane 100% *w*/*v*, Zoetis, Parsippany-Troy Hills, NJ, USA); Fluorescein (Fluoralfa, Alfa Intes, Casoria, Naples, Italy); TRI Reagent (Thermo Fisher Scientific, Waltham, MA, USA); iScript™ cDNA Synthesis Kit (Bio-Rad Laboratories, Hercules, CA, USA); SsoAdvanced™ Universal SYBR^®^ Green Supermix (Bio-Rad Laboratories, Hercules, CA, USA) and QuantStudio™ 5 Real-Time PCR System (Thermo Fisher Scientific, Whaltam, MA, USA); hematoxylin–eosin (Harris’s Hematoxylin, Cat. No. 01HEMH2500; Eosin, Cat. No. 01EOY101000; Histo-Line Laboratories, Pantigliate, MI, Italy); Alcian blue (pH 2.5; Cat. No. 04-160802; Bio-Optica, Milan, Italy); periodic acid–Schiff (PAS, Cat. No. 04-130802; Bio-Optica, Milan, Italy); BSA (Cat. No. A9418; Sigma-Aldrich, Darmstadt, Germany); Tween-20 (Cat. No. P9416; Sigma-Aldrich); biotinylated anti-rabbit IgG secondary antibody (Vector Laboratories, Newark, CA, USA); avidin–biotin–peroxidase complex (VECTASTAIN ABC Kit, Peterborough, UK); Complement C3 Polyclonal Antibody (rabbit polyclonal antibody, Cat. No. PAS-21349; Invitrogen, Rockford, IL, USA); Complement C5a Polyclonal Antibody (rabbit polyclonal antibody, Cat. No. PAS-78891; Invitrogen, Rockford, IL, USA), optical microscope (Eclipse E600; Nikon, Shinjuku, Japan); Imaging Source “33” Series USB 3.0 camera (Cat. No. DFK 33UX264; Bremen, Germany); Grundium Ocus 20 scanner (Tampere, Finland); ImageJ software (version 1.53a; National Institutes of Health, Bethesda, MD, USA); Controlled-Environment Chamber (CEC) (Laboratory Products Inc., Seaford, DE); GraphPad Prism software (version 10.0 or 8.0.2; GraphPad Software, San Diego, CA, USA); QuPath 0.6.0 software (Free Open Source software).

### 2.2. Animals

All experiments were performed using female C57BL/6J mice, aged 12 weeks at the beginning of the study. Animals were used only after a 7-day acclimatization period. During the acclimatization period and during phases outside the controlled-environment chamber (CEC), mice were housed in open cages with corncob bedding to reduce dust exposure and provided with environmental enrichment, in accordance with standard animal welfare guidelines. Throughout the entire housing period, including the experimental phase, animals were maintained under a 12 h light/12 h dark cycle and had ad libitum access to food and water.

### 2.3. Induction and Dry Eye Model Procedure

Induction of the DED model requires a CEC that guarantees specific conditions of airflow, humidity, and temperature, as previously described [13]. Mice were placed in a controlled environmental room (temperature: 20–22 °C; relative humidity: 25%; airflow: 15 L/min) and the mice were then randomly assigned to 3 groups (*n* = 5 per group), including an untreated group (CTR) in which the mice received no induction.

### 2.4. Experimental Design

Throughout all experimental procedures, animals had free access to food and water. Prior to exposure to CEC, corneal integrity in both eyes was evaluated by fluorescein instillation and examination under cobalt blue illumination. Mice presenting marked corneal damage before DED induction were excluded from the study and replaced. Pre-injury assessments (Baseline Score) were conducted to evaluate corneal integrity. Animals were then randomly assigned to the experimental groups and divided into two groups of mice (*n* = 10) exposed to the CEC: one exposed group (EG) for 14 days (14EG) and one EG for 21 days (21EG).

In this DED model, mice were housed in the CEC for 12 h per day over 14 consecutive days. Control animals consisted of age-matched mice maintained under standard housing conditions (relative humidity 50–80%, no airflow, temperature 21–23 °C) and are referred to throughout this study as controls (CTR).

Deeply isoflurane-anesthetized animals were euthanized after 14 and 21 days from the beginning of induction, as well as the CTR animals. The eye globes, including both superior and inferior lids, were excised, washed in cold PBS pH 7.4 to remove excess blood, and tissues were further processed as described below.

### 2.5. Ocular Surface Evaluation—Corneal Fluorescein Staining

To evaluate corneal epithelial damage over time, mice were examined at three experimental time points (Baseline, 14 days, 21 days) while under isoflurane anesthesia. Corneal epithelial damage was assessed using topical sodium fluorescein staining. Fluorescein is a low-molecular-weight hydrophilic dye that does not penetrate an intact corneal epithelium; under physiological conditions, tight junctions and the mucin-rich glycocalyx limit its paracellular diffusion. In the presence of desiccating stress-induced barrier disruption, characterized by increased epithelial permeability, tight junction alteration, and surface mucin loss, fluorescein accumulates within intercellular spaces and areas of epithelial discontinuity, producing the characteristic punctate staining pattern indicative of ocular surface damage [25].

Ocular surface assessment was performed by slit-lamp biomicroscopy under cobalt blue illumination following topical fluorescein staining. Specifically, 0.5 µL of a 0.2% fluorescein solution (Fluoralfa; Alfa Intes, Casoria, Naples, Italy) was instilled into the lower conjunctival sac of both eyes using a micropipette. After 3 min uptake period, excess dye was carefully removed from the lateral canthus. Corneal regions exhibiting fluorescein accumulation were identified on the acquired images (one image per eye per time point) and quantitatively analyzed. The corneal staining ratio was calculated as the ratio between the fluorescein-positive corneal area and the total corneal surface and is expressed as a percentage. The severity of corneal fluorescein staining was assessed in a blinded manner using the National Eye Institute (NEI) grading system, which assigns a score from 0 to 3 to each of five corneal regions (central, superior, inferior, nasal, and temporal), providing a semi-quantitative evaluation of staining severity [26]. All image analyses were performed using ImageJ software (version 1.53a; National Institutes of Health, Bethesda, MD, USA).

### 2.6. Histology and Immunohistochemistry

**Figure 1:**
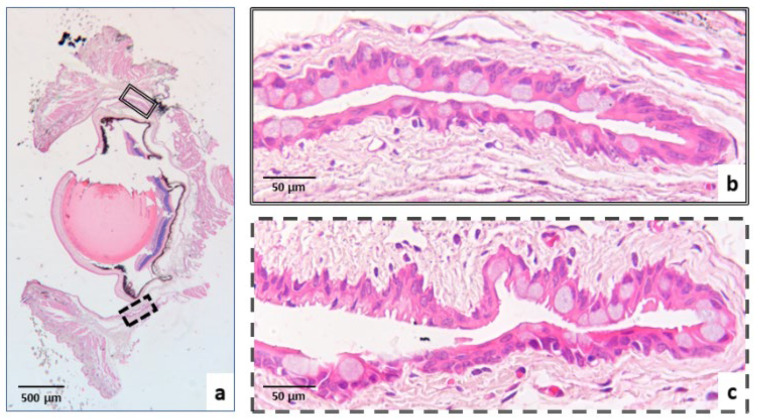
Representative histological sections of the ocular globe: (**a**) Central vertical plane at 4× magnification, stained with hematoxylin and eosin, showing the two regions of interest (ROIs); (**b**) Superior fornix at 40× magnification (double-lined box in (**a**)); (**c**) Inferior fornix at 40× magnification (dashed box in (**a**)).

The specimens (the right eye of each mouse) assigned to morphological analysis for histological and immunohistochemical evaluation were fixed in 4% buffered paraformaldehyde and stored at room temperature until processing. After fixation, tissues were dehydrated through an ascending ethanol series and embedded in paraffin. Four 3-μm-thick sections obtained from the central vertical plane of the ocular globe were stained with hematoxylin and eosin to evaluate conjunctival epithelial and GC morphology, with Alcian–periodic acid–Schiff (PAS), a histochemical stain that allows visualization of neutral (PAS-positive) and acidic (Alcian blue-positive) mucins, to assess changes in neutral and acidic mucins in GCs; and by immunohistochemistry using anti-C3 and anti-C5a antibodies to quantify complement fractions. The target areas of investigation in each eye were the superior and inferior fornices of the eyelids (Figure 1).

Two groups of animals were compared for histological analysis: control mice (CTR), consisting of five mice not exposed to dry-eye induction; their values were considered baseline; exposed group (EG) mice, representing the dry-eye induction model: five mice sacrificed after 14 days of induction (14EG) and five mice sacrificed after 21 days of induction (21EG).

The histological variables investigated included GCnumber, size, and area, assessed by morphometric analysis; changes in GCmucin content, assessed by histochemistry; and C3 and C5a levels, assessed by immunohistochemistry.

#### 2.6.1. Evaluation of GC Number, Size and Area

After hematoxylin–eosin staining (Harris’s Hematoxylin, Cat. No. 01HEMH2500; Eosin, Cat. No. 01EOY101000; Histo-Line Laboratories, Pantigliate, MI, Italy), images of the superior and inferior fornices of the ocular globes were acquired using an optical microscope (Eclipse E600; Nikon, Shinjuku, Japan) equipped with an Imaging Source “33” Series USB 3.0 camera (Cat. No. DFK 33UX264; Bremen, Germany) at 2.5×, 4×, 10×, 20×, 40× and 63× magnification.

Image analysis was performed using ImageJ software. After calibrating the scale bar according to the magnification used, measurements were collected only when the fornix was entirely evaluable. Images acquired at 4× magnification were used to measure the total length of the superior and inferior fornices using the ImageJ “straight” length measurement tool; values were expressed in micrometers. At 10× and 20× magnification, all GCs present along both fornices were counted using the ImageJ cell counter tool. At 40× magnification, measurements were obtained from 10 randomly selected GCs per fornix; for each cell, the major and minor diameters were measured.

Collected data were processed in an Excel file. For each case, GC counts were standardized to 1500 µm of fornix length, and the major and minor diameters of GCs were averaged separately for each fornix. Statistical analyses were performed using, for each case, the following parameters: the number of GCs per 1500 µm (calculated as the mean between the superior and inferior fornices), the mean major diameter, and the mean minor diameter of GCs obtained by averaging the measurements from both fornices.

The quantification of the GC coverage area in the fornix was performed by digitizing slides with a Grundium Ocus 20 scanner (Tampere, Finland), which generated whole-slide images (WSIs). Advanced image analysis was conducted using QuPath 0.6.0 software [27]. For each case, the GC area was measured within a selected region corresponding to a 1500 µm length in each fornix. GC area quantification was obtained using pixel classification tools based on chromatic recognition. The GC classifier was generated using the Random Trees algorithm at high spatial resolution (0.50 µm/px), with training based on 15 manual annotations per marker to differentiate GCs from other cells located in the fornix. The area occupied by GCs (µm^2^) was extracted, and the mean area for each case was calculated.

#### 2.6.2. Mucin Variations Assessed by Histochemistry

Alcian–PAS staining was employed to assess variations in neutral (PAS-positive) and acidic (Alcian blue-positive) mucins in GCs. Briefly, Alcian blue (pH 2.5; Cat. No. 04-160802, Bio-Optica, Milan, Italy) was applied first, followed by periodic acid–Schiff (PAS; Cat. No. 04-130802, Bio-Optica, Milan, Italy). This combined staining allowed evaluation of GC mucin composition, specifically highlighting non-sulfated acidic mucins (stained blue by Alcian blue at pH 2.5) and neutral mucins (stained magenta by PAS), while a mixture of neutral and acidic mucins produced a purple staining. GCs with cytoplasm stained blue, magenta, or purple were counted in both the superior and inferior fornices using an optical microscope (Eclipse E600; Nikon, Shinjuku, Japan) equipped with a cell counter application. Data were expressed as the percentage of blue-, magenta-, and purple-stained GCs per 1500 µm.

#### 2.6.3. C3 and C5a Immunohistochemistry

Immunohistochemistry was performed on two serial 3 µm thick sections to assess C3 and C5a expression. Sections were dewaxed and rehydrated, and endogenous peroxidase activity was blocked by immersion in 0.3% H_2_O_2_ in methanol for 30 min at room temperature. Heat-induced antigen retrieval was performed only for the anti-C3 antibody by heating the sections in EDTA buffer (pH 8.0) for two cycles of 5 min each, in a microwave oven at 750 W; antigen retrieval was not performed for the anti-C5a antibody. Sections were then allowed to cool to room temperature for 20 min. For each protocol, tissue sections were incubated overnight at 4 °C in a humidified chamber with primary antibodies against C3 (rabbit polyclonal antibody, Cat. No. PAS-21349; Invitrogen, Rockford, IL, USA) or C5a (rabbit polyclonal antibody, Cat. No. PAS-78891; Invitrogen, Rockford, IL, USA), both diluted 1:200 in a solution containing 3% BSA (Cat. No. A9418; Sigma-Aldrich, Darmstadt, Germany) and 0.25% Tween-20 (Cat. No. P9416; Sigma-Aldrich) in PBS (0.01 M, pH 7.2). Binding sites were detected using a biotinylated anti-rabbit IgG secondary antibody (Vector Laboratories, Newark, CA, USA; 1:200 dilution in blocking solution, 30 min at room temperature) and amplified with a commercial avidin–biotin–peroxidase complex (VECTASTAIN ABC Kit, Peterborough, UK). The chromogen 3,3′-diaminobenzidine (0.05%) was used, and slides were counterstained with Harris hematoxylin. Immunoreactivity was observed only in fornix epithelial cells, with no staining detected in GCs. Staining was scored semiquantitatively according to the percentage of positively stained epithelial cells using a four-point scale: score 1, no positivity (0% positive cells); score 2, low positivity (<40% positive cells); score 3, moderate positivity (40–60% positive cells); and score 4, high positivity (70–100% positive cells).

### 2.7. Molecular Detection of Inflammatory Markers—Molecular Biology

#### RNA Extraction and cDNA Reverse Transcription

Given the leading role of inflammation in the pathogenesis of DED, the expression of selected inflammatory markers was analyzed in ocular tissues, in particular Major Histocompatibility Complex, Class II, DR (HLA-DR), IL-1β, TNF-α, and LTB4.

To conduct the molecular biology analyses, the excised samples were sectioned in order to isolate the cornea and conjunctiva from the eye globe.

Total RNA extraction was performed by using TRIzol™ Reagent (Thermo Fisher Scientific, Waltham, MA, USA) according to the manufacturer’s instructions. The phenol-chloroform extraction procedure was used for total RNA extraction from dissected ocular tissues. RNA was precipitated with isopropanol and washed in ethanol before reconstitution in nuclease-free water. Two-microliter RNA samples were quantified using the NanoDrop ND-1000 spectrophotometer (Thermo Fisher Scientific, Waltham, MA, USA) and one μg of total RNA was reverse transcribed in a 20 μL reaction volume using the iScriptTM cDNA synthesis kit (Bio-Rad Laboratories, Hercules, CA, USA) following the manufacturer’s instructions.

RT-PCR analysis was carried out on a QuantStudio™ 5 Real-Time PCR System (Applied Biosystems^®^ by Thermo Fisher Scientific, Waltham, MA, USA), using the semi-quantitative Sybr Green (SsoAdvancedTM Universal Sybr Green Supermix; Bio-Rad Laboratories, Hercules, CA, USA) approach.

The assay was executed in triplicate and target gene expression was normalized to β-actin (mouse—*Mus musculus*), which was used as the housekeeping gene. The final results were determined by the comparative 2^−ΔΔCt^ method and expressed as fold changes relative to controls. Specific pairs of primers (Merck Group, Darmstadt, Germany) were designed by using the NCBI Blast Tool (Table 1).

### 2.8. Statistical Analysis

All experiments were performed with at least three biological and technical replicates. Data are presented as mean ± standard deviation (SD), unless otherwise specified. Statistical analyses and graphical representations were performed using GraphPad Prism software (version 10.0 or 8.0.2; GraphPad Software, San Diego, CA, USA). For molecular biology analyses, comparisons among multiple groups were carried out using ordinary one-way analysis of variance (ANOVA) followed by Tukey’s post hoc test. For histopathological analyses, data distribution was first assessed using the Shapiro–Wilk normality test. Normally distributed data were analyzed using unpaired *t*-tests (for two-group comparisons) or one-way ANOVA, whereas non-normally distributed data were analyzed using the Mann–Whitney U test or Kruskal–Wallis test followed by appropriate multiple-comparison procedures. Results from histopathological analyses are presented as box plots showing mean ± SD or median, as appropriate. When no statistically significant differences were detected between the 14EG and 21EG groups, data were pooled and compared with the control group (CTR). Conversely, when significant differences were observed, comparisons were performed among the CTR, 14EG, and 21EG groups. In all analyses, differences were considered statistically significant at *p* < 0.05.

## 3. Results

### 3.1. Corneal Damage Assessment

**Figure 2:**
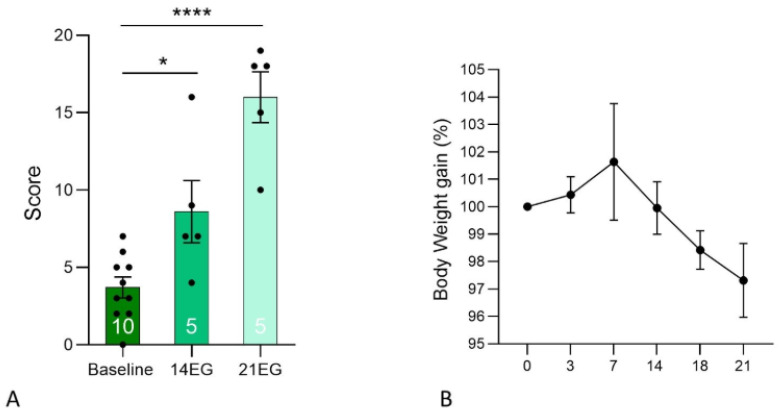
(**A**) Quantitative analysis of corneal fluorescein staining at baseline, 14 days (14EG), and 21 days (21EG). Baseline measurements include the entire cohort (*n* = 10), which was subsequently divided and re-evaluated at 14 days (*n* = 5) or 21 days (*n* = 5). Statistical comparisons between baseline and each time point were performed (* *p* = 0.0117 at 14 days; **** *p* < 0.0001 at 21 days). (**B**) Body weight changes over the experimental period, expressed as percentage difference from baseline.

Corneal damage was assessed by fluorescein staining and quantitatively analyzed at baseline and at subsequent experimental time points. At baseline, a total of 10 animals were evaluated, corresponding to the combined cohort later assigned to the 14EG and 21EG experimental groups. Following baseline assessment, animals were randomly divided and re-evaluated at 14 days (*n* = 5) or 21 days (*n* = 5). Statistical analysis was performed using Student’s *t*-test, comparing baseline values with those obtained at each time point. A significant increase in corneal damage was observed at 14 days compared with baseline (*p* = 0.0117). An even more pronounced increase was detected when baseline values were compared with those measured at 21 days (*p* < 0.0001), indicating a progressive and sustained worsening of corneal surface integrity over time (Figure 2A). Body weight was monitored throughout the experimental period as an indicator of animal welfare and general health status. As shown in Figure 2B, animals exhibited only minimal variations in body weight during the induction period, expressed as percentage change from individual baseline values. Although a slight decrease in body weight was observed, consistent with mild discomfort associated with the induction procedure, weight loss never exceeded 5% of baseline, indicating limited distress and good overall tolerability of the experimental protocol. Individual body weight data are reported in Supplementary Table S1.

### 3.2. Histology, Histochemistry and Immunohistochemistry

#### 3.2.1. Evaluation of GC Number and Size

**Figure 3:**
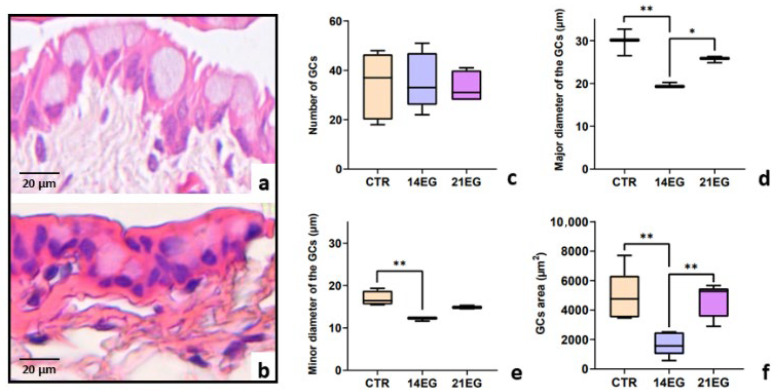
Evaluation of GC number, size and area. In (**a**), morphological features of the epithelium and GCs in the fornix of mice from the control group. In (**b**), the same site shows densely packed cells with deeply stained cytoplasm and a reduction in the size of the GCs. The graphs (median, min and max values; * *p* < 0.05; ** *p* < 0.01) show variations between the exposed groups in the number of GCs (**c**), in their major (**d**) and minor (**e**) diameters and coverage area (**f**).

Histological analysis of the lid fornix did not reveal any significant changes in the epithelium or in the number of GCs (*p*-value always >0.05) (Figure 3a–c), but only variations in GC size. Analysis of both the major and minor diameters of GCs, obtained through image analysis (normally distributed according to the Shapiro–Wilk test), showed, comparing the CTR group with the 14EG and 21EG groups using one-way ANOVA with multiple-comparisons testing, a significant reduction in the mean GC diameter in the 14EG group (*p* = 0.0014 for the major diameter) (Figure 3d); (*p* = 0.0029 for the minor diameter) (Figure 3e). However, no significant difference was observed between the CTR and 21EG groups for both diameters; instead, the 21EG group showed a slight increase in the major diameter. This slight increase in GC size in the 21EG group was confirmed by a statistically significant difference when compared with the 14EG group (*p* = 0.0174 for the major diameter) (Figure 3d). A further confirmation that the reduction in GC size is a hallmark of this disease model comes from the analysis of the GC coverage area in the fornix. Analysis of GC-covered area, whose data were normally distributed according to the Shapiro–Wilk test, comparing the CTR group with the 14EG and 21EG groups using one-way ANOVA with multiple comparisons, revealed a significant reduction in GC area in the 14EG group (*p* = 0.0053) (Figure 3f). However, similarly to the evaluation of GC diameters, no statistically significant differences were observed between the CTR and 21EG groups. The 21EG group showed an increase in the area occupied by GCs, confirmed by a statistically significant difference compared with the 14EG group (*p* = 0.0086) (Figure 3f).

#### 3.2.2. Evaluation of Mucin Variations Revealed by Histochemistry

**Figure 4:**
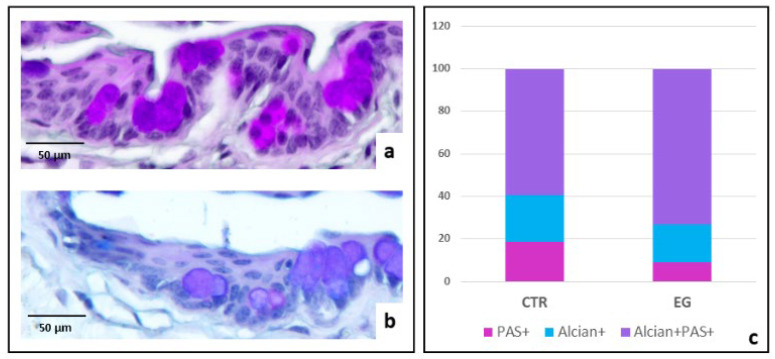
Mucin variations assessed by Alcian-PAS staining. Alcian–PAS staining in CTR (**a**) shows a higher number of magenta-stained GCs (neutral mucins) compared with EG (**b**), where they appear reduced and mainly express a mixed acidic and neutral mucin content (purple staining). In (**c**), the percentage of GCs containing neutral or acidic non-sulfated mucins (blue staining), or their mixture, in the two experimental groups is shown. In the EG, the number of magenta-positive GCs (containing neutral mucins) is decreased compared with the control group.

The comparison between the 14EG and 21EG groups in the number of GCs containing exclusively acidic non-sulfated mucins (blue staining), neutral mucins (magenta staining), or a mixture of both (purple staining) did not reveal any statistically significant differences. Therefore, histochemical data from GCs were pooled and analyzed as a single exposed group (EG). The comparison between the CTR and the EG showed that the number of GCs containing PAS-positive neutral mucins was significantly lower in the EG than in the CTR (Figure 4c, *p* = 0.0019, Chi-square with Yates correction).

#### 3.2.3. Evaluation of C3 and C5a Expression Assessed by Immunohistochemistry

**Figure 5:**
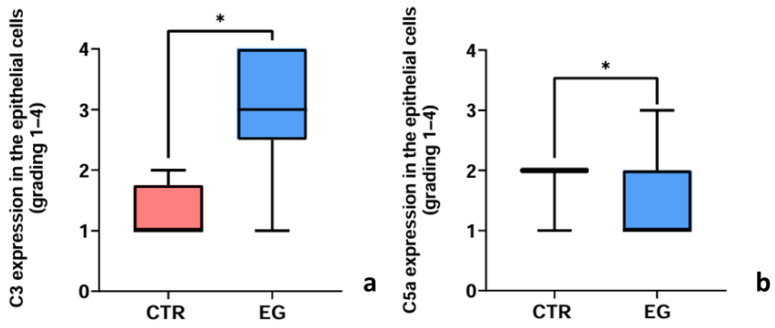
C3 and C5a immunohistochemistry expression. Comparison of (**a**) C3 and (**b**) C5a scores between the two experimental groups shows that the median score is higher in the EG group than in the CTR (* *p* < 0.05).

Immunohistochemical staining for anti-C3 and anti-C5a antibodies was graded according to staining intensity. Immunoreactivity was detected in epithelial cells of the fornix but was never observed in GCs. Representative examples of both C3 and C5a staining are shown in Supplementary Figure S1. The comparison between the 14EG and 21EG groups for the immunohistochemical expression scores of C3 and C5a did not reveal any statistically significant differences. Therefore, immunohistochemical data were pooled and analyzed as a single exposed group (EG). The graphs in Figure 5 show the results of the semiquantitative assessment (median, min and max values), which was significantly higher in the EG compared with the control group (CTR) for both C3 (Figure 5a) and C5a (Figure 5b), as assessed by the Mann–Whitney test (*p* = 0.0381 for C3; *p* = 0.0479 for C5a).

### 3.3. Determination of Inflammatory Markers—Molecular Biology

**Figure 6:**
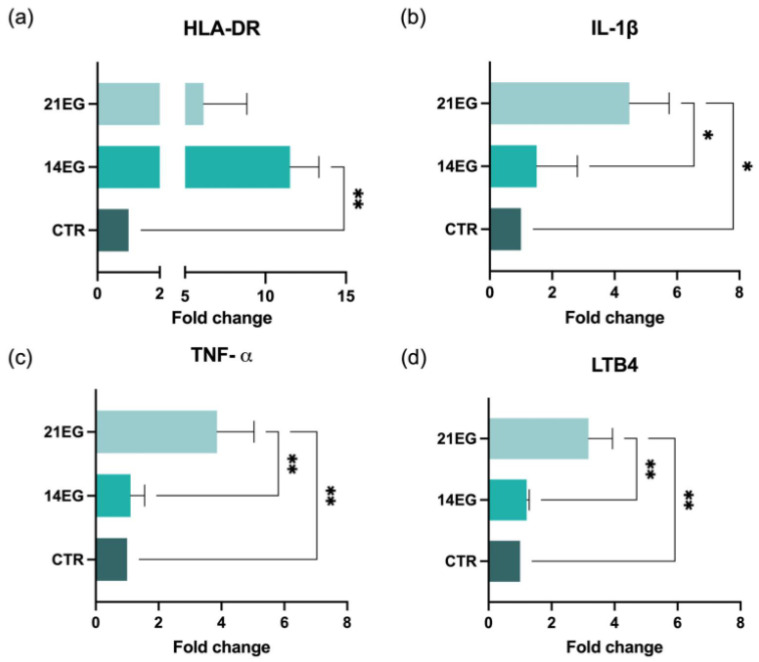
Results for the inflammatory markers (**a**) HLA-DR, (**b**) IL-1β, (**c**) TNF-α and (**d**) LTB4 expression in the control group (CTR) and exposed groups for 14 (14EG) and 21 days (21EG). Cycle threshold (Ct) values were normalized to the Actin-β gene, and the final data are reported as fold relative to untreated controls and mean ± SD of three biological replicates. Significance * *p* < 0.05; ** *p* < 0.01 (Ordinary one-way ANOVA, followed by Tukey’s multiple comparison tests).

Results for the detection of inflammatory markers are summarized in Figure 6.

Compared with control mice (CTR), animals exposed to desiccating stress showed a significant increase in HLA-DR expression at 14 days after induction (Figure 6a), followed by a marked reduction at 21 days. Similarly, the pro-inflammatory cytokines IL-1β (Figure 6b) and TNF-α (Figure 6c) were significantly elevated in induced mice compared to CTR animals, with higher expression levels observed at the later time point. In parallel, the expression of leukotriene B4 (LTB4) (Figure 6d) was significantly increased following dry eye induction, displaying a time-dependent trend. Overall, these molecular findings demonstrate progressive activation of inflammatory pathways in response to desiccating stress, supporting the establishment of a stable dry eye phenotype in this model.

## 4. Discussion

Preclinical animal models are indispensable tools for elucidating disease mechanisms and for evaluating therapeutic strategies [28,29]. Several foundational murine models have been developed, including aqueous-deficient models (e.g., lacrimal gland excision) and pharmacologically induced models combining controlled environmental desiccating stress with cholinergic blockade (e.g., scopolamine) to suppress lacrimation and promote DED features [30,31]. Although tear secretion tests such as the phenol red thread assay are frequently used in aqueous-deficient models, environmentally induced desiccating stress models primarily affect tear film stability and inflammatory pathways rather than directly suppressing lacrimal output [32]. For this reason, epithelial damage and molecular inflammatory markers may represent more consistent indicators of disease induction in such settings. Environmental stress models have been recently examined in controlled humidity chambers, providing an opportunity to mimic evaporative forms of DED that closely resemble naturally occurring disease in humans [29,33]. In the present study, a desiccation-only protocol was employed to induce DED-like features in mice over a 21-day period. This approach yielded progressive and sustained corneal surface damage, as evidenced by significant increases in fluorescein staining at 14 and 21 days relative to baseline. These findings align with previous reports demonstrating that controlled environmental stress is sufficient to disrupt tear film homeostasis and induce epithelial damage in rodents, even in the absence of pharmacological lacrimal inhibition [29]. A major advantage of the desiccation-only model is its limited impact on general animal welfare, as indicated by minimal changes in body weight that did not exceed the 5% threshold commonly used to gauge significant distress. This observation suggests that the corneal pathology induced in this model arises predominantly from ocular surface stress rather than systemic distress, making it a more humane and specific tool for studying evaporative DED while adhering to refinement principles in animal research.

GCs were a target of investigation in the disease model we propose. We observed a significant reduction in cell size—possibly due to reduced hydration of the ocular conjunctiva—and a significant decrease in neutral mucins. The reduction in size parallels observations by Khimani et al. (2020), who reported that in the aqueous tear-deficient form of the spontaneous human pathology, GC area is a better disease measure than GC number [34]. In the eye, GCs are the principal secretory cells of the conjunctival epithelium. They lubricate the ocular surface during blinking, stabilize the tear film, and act as a physical barrier against pathogen penetration [17,34,35]. The primary function of GCs is mucin production, with a steady basal level of exocytosis [36]. Upon detection of potential threats, however, a burst of secretion occurs, entrapping pathogens and debris to facilitate their removal. Variations in mucin exocytosis are associated with changes in mucin type under chronic mechanical or inflammatory stimuli [37]. Alterations in mucin glycosylation, including increased sialylation—which enhances the acidic nature of mucins—have been reported in ocular surface pathologies such as dry eye [38], contact lens-related pathology [39], pterygium [37], and ocular rosacea [40], as well as in the airway [41], gastrointestinal tract [42], and cancer [43]. Matsuzawa et al. (2023) demonstrated that Alcian blue staining in GCs of C57BL/6 mice reflects mucin sialylation [37]. In this strain, as in humans, staining correlates with sialyltransferase activity, which increases under pathological conditions. Sialylated mucins represent a key ocular defense mechanism, as they improve particle capture and enhance clearance. Alcian blue–PAS staining in the proposed model revealed, in EG mice, a significant reduction in GCs containing neutral mucins, possibly due to a shift toward increased production of acidic mucins, consistent with what occurs in irritative ocular conditions.

This pattern is consistent with adaptive responses described in other mucosal tissues exposed to chronic environmental stress, such as the nasal and airway epithelium, where GCs initially undergo plastic remodeling of secretory activity and cellular volume before overt cell loss occurs [34,44,45,46].

Furthermore, inflammation—demonstrated by significantly higher levels of cytokines and complement fractions in EG compared with CTR mice—is another cause of GC dysfunction. TNF-α and interferon-γ (IFN-γ) are known to induce apoptosis and inhibit cholinergically stimulated mucin secretion [35]. Thus, the objective evidence of inflammation in the proposed model may directly impair conjunctival GC function, resulting in altered tear composition and compromised protective capacity, thereby contributing to ocular surface damage in this dry eye mouse model.

Multiple animal models of DED have been used to elucidate its pathophysiology and to develop novel treatments. These models use mice, rats, rabbits, cats, dogs and non-human primates. Each model assesses aspects of DED by focusing on elements of the lacrimal functional unit, which controls the homeostasis of the tear film. No comprehensive ‘ideal’ animal model encompassing all aspects of human DED exists nor is it feasible [47].

The temporal modulation of inflammatory markers observed in this study further supports the validity of the murine DED model. HLA-DR expression has been widely reported as elevated in DED, reflecting enhanced antigen presentation and immune activation at the ocular surface due to desiccating stress and tear film instability [48,49]. The early increase in HLA-DR expression at 14 days is consistent with the activation of antigen-presenting cells typically associated with acute ocular surface inflammation. While experimental models of DED typically show upregulation of inflammatory mediators, the temporal dynamics of these responses can vary depending on the specific marker and phase of disease progression.

However, sustained HLA-DR expression during prolonged exposure may reflect a chronic stress-induced activation state that engages immunoregulatory mechanisms aimed at modulating the immune response and limiting excessive tissue damage [50]. The subsequent reduction in HLA-DR expression at 21 days suggests the engagement of adaptive or regulatory mechanisms that limit prolonged immune activation.

In contrast, pro-inflammatory cytokines such as IL-1β, TNF-α, and lipid mediators like LTB4 are often persistently elevated in both human and animal DED models, contributing to continued cytokine signaling and chronic inflammation [51].

IL-1β and TNF-α are key mediators of ocular surface inflammation and have been widely implicated in epithelial damage, barrier dysfunction, and tear film instability in DED [50]. Similarly, LTB4, a potent lipid mediator involved in leukocyte recruitment and amplification of inflammatory responses, has been reported to contribute to chronic inflammation at the ocular surface [52]. Together, these findings suggest that while certain immune components such as HLA-DR may undergo temporal regulation, other inflammatory pathways persist during prolonged desiccating stress, supporting the establishment of a chronic inflammatory milieu characteristic of DED.

Recent epidemiological studies have increasingly emphasized the impact of environmental conditions and lifestyle factors on ocular surface homeostasis and the prevalence of DED. While such investigations provide important real-world evidence, controlled experimental models remain essential to elucidate the underlying mechanisms under defined conditions. In this context, the present study offers an environmentally relevant and mechanistically informative platform for translational and preclinical research.

## 5. Conclusions

Chronic desiccating stress induced progressive corneal epithelial damage, conjunctival GC remodeling with altered mucin composition, and significant activation of inflammatory and complement pathways. The upregulation of HLA-DR, IL-1β, TNF-α, LTB4, C3, and C5a confirms the establishment of a sustained inflammatory ocular surface phenotype consistent with key features of human evaporative DED. Overall, the CEC-based murine model provides a clinically relevant and controlled platform for investigating environmentally driven ocular surface inflammation and for the preclinical evaluation of targeted therapeutic strategies.

## Figures and Tables

**Table 1:** List and Sequence of Primers Used for RT-PCR.

Gene	Forward Primer Sequence (5′-3′)	Reverse Primer Sequence (3′-5′)
*β-actin*	GCAAGCAGGAGTACGATGAGT	AGGGTGTAAAACGCAGCTCAG
*TNF-α*	ACTGAACTTCGGGGTGATCG	TGGTGGTTTGTGAGTGTGAGG
*IL-1β*	TGCCACCTTTTGACAGTGATG	TTGGAAGCAGCCCTTCATCTT
*LTB4*	GGACCCTGGCACTAAGACAG	AGCCATCAAAAGGACAGGGTT
*HLA-DR*	TTTACGACTGCAGGGTGGAG	AGGGCTTGGAGCATCAAACT

*IL-1β*: interleukin-1β; *TNF-α*: tumor necrosis factor-α; *HLA-DR*: Major Histocompatibility Complex, Class II, DR; *LTB4*: Leukotriene B4.

## Data Availability

The data presented in this study are available upon well motivated request from the corresponding author.

## Supplementary Materials

The following supporting information can be downloaded at: https://www.mdpi.com/article/10.3390/biomedicines14030693/s1, Figure S1: Representative examples of both C3 and C5a staining for each score positivity value: score 1, no positivity (0% positive cells); score 2, low positivity (<40% positive cells); score 3, moderate positivity (40–60% positive cells); and score 4, high positivity (70–100% positive cells); Table S1: Mice body weight (in g.) monitoring during the experimental period.

## Abbreviations

The following abbreviations are used in this manuscript:

ARRIVE	Animal Research Reporting of In Vivo Experiments
cDNA	Complementary Deoxyribonucleic Acid
CEC	Controlled Environmental Chamber
Ct	Cycle threshold
CTR	Control
DED	Dry Eye Disease
EDTA	Ethylenediaminetetraacetic Acid
EG	Exposed Group
GC	Goblet Cell
H_2_O_2_	Hydrogen Peroxide
HLA-DR	Major Histocompatibility Complex, Class II, DR
IFN-γ	Interferon-γ
IL-1	Interleukin-1
IL-1β	Interleukin-1β
LTB4	Leukotriene B4
MMP-9	Matrix Metalloproteinase-9
NEI	National Eye Institute
PAS	Periodic-Acid Shiff
RNA	Ribonucleic Acid
ROI	Region of Interest
RT-PCR	Real-Time PCR
SD	Standard Deviation
TFOS DEWS	Tear Film & Ocular Surface Society Dry Eye Workshop
TNF-α	Tumor Necrosis Factor-α
WSIs	Whole-Slide Images
