# Supplementary material for: Integrated Evaluation of Corneal Damage, Goblet Cell Remodeling and Inflammatory Response in a Murine Model of Environmental Dry Eye Disease (DED)
Source: Biomedicines. 2026 Mar 17;14(3):693. doi: 10.3390/biomedicines14030693 (PMC13024243; doi:10.3390/biomedicines14030693)
Supplement: Supplementary file 1 [file biomedicines-14-00693-s001.zip › R1_Supplementary Materials - Table S1.pdf]

Supplementary Materials

Supplementary Table S1: Mice body weight (in g.) monitoring during the experimental period.

Group	Day 0	Day 3	Day 7	Day 14	Day 18	Day 21
14 EG	20,2	19,8	20	19,8	-	-
	20,3	20,3	20,4	19,8	-	-
	22	22	21,9	21,4	-	-
	20,7	20,9	21	20,5	-	-
	20,8	20,9	21,1	20,8	-	-
21 EG	22,4	22,2	19,9	22,3	22,1	21,6
	19,9	20,1	22,5	20,4	19,2	18,8
	22,3	22	21,8	21,5	21,3	21,4
	22,6	22,7	23,6	23,4	23,3	22
	21,2	22,4	23,4	22,4	21,3	21,7

EG: Exposed Group
