# Supplementary material for: Integrated Evaluation of Corneal Damage, Goblet Cell Remodeling and Inflammatory Response in a Murine Model of Environmental Dry Eye Disease (DED)
Source: Biomedicines. 2026 Mar 17;14(3):693. doi: 10.3390/biomedicines14030693 (PMC13024243; doi:10.3390/biomedicines14030693)

Supplementary Materials

Supplementary Figure S1: Representative examples of both C3 and C5a staining for each score positivity value: score 1, no positivity (0% positive cells); score 2, low positivity (<40% positive cells); score 3, moderate positivity (40–60% positive cells); and score 4, high positivity (70–100% positive cells).

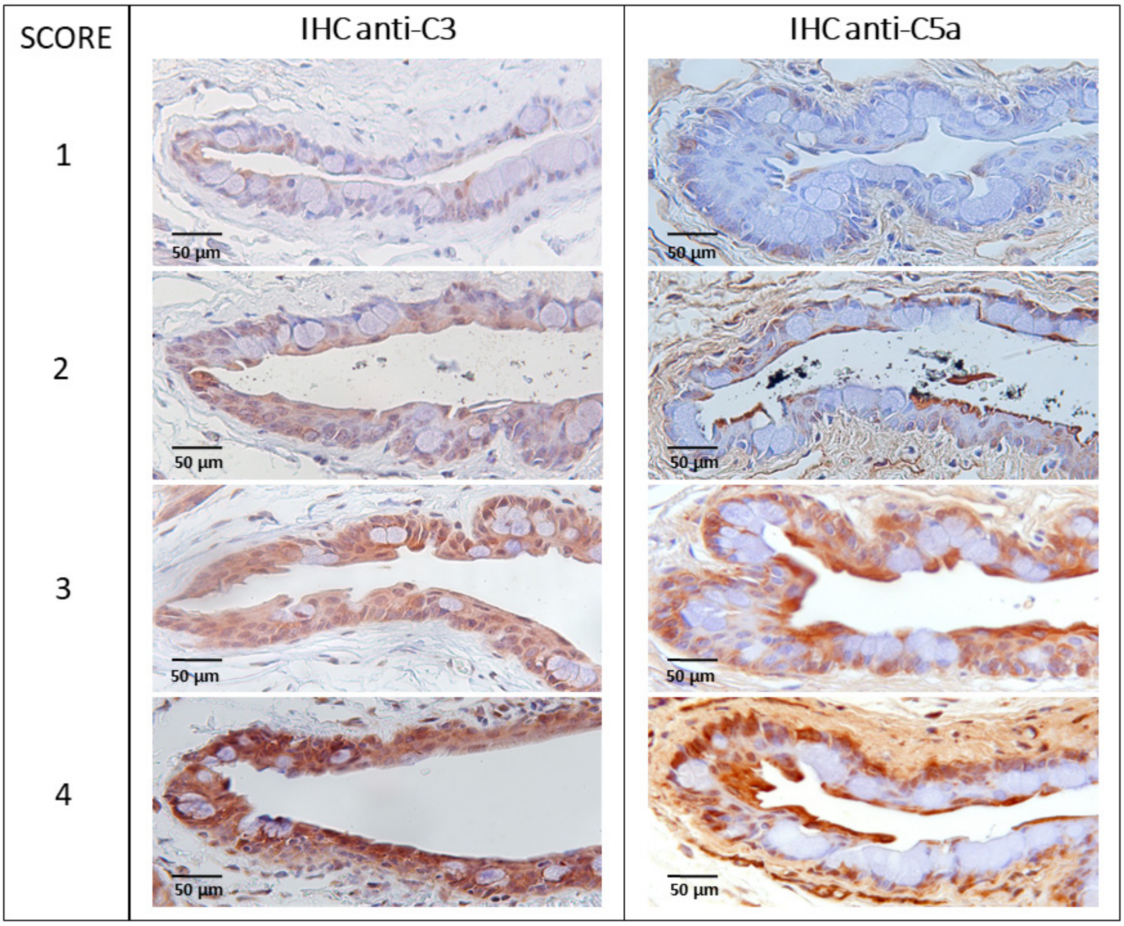

Supplement: Supplementary file 1 [file biomedicines-14-00693-s001.zip › R2_Supplementary Materials - Figures.pdf]
